# RIPK3 Contributes to Thyroid Hormone-Induced Photoreceptor Degeneration †

**DOI:** 10.3390/ijms26178154

**Published:** 2025-08-22

**Authors:** Lilliana R. York, Hongwei Ma, Yun Le, Courtney T. Griffin, Xi-Qin Ding

**Affiliations:** 1 Department of Cell Biology, University of Oklahoma Health Sciences, Oklahoma City, OK 73104, USA; lilliana-york@ou.edu (L.R.Y.); hongwei-ma@ou.edu (H.M.); courtney-griffin@omrf.org (C.T.G.); 2 Departments of Medicine Endocrinology, Cell Biology, and Ophthalmology, and Harold Hamm Diabetes Center, University of Oklahoma Health Sciences, Oklahoma City, OK 73104, USA; 3 Cardiovascular Biology Research Program, Oklahoma Medical Research Foundation, Oklahoma City, OK 73104, USA

**Keywords:** photoreceptor, retina, thyroid hormone, RIPK3, necroptosis

## Abstract

Thyroid hormone (TH) regulates cell proliferation, differentiation, and metabolism. Increased TH levels in circulation are associated with a higher incidence of age-related macular degeneration. In mice, TH treatment causes photoreceptor degeneration, which is accompanied by an increase in receptor-interacting serine/threonine-protein kinase 3 (RIPK3) in the retina. Here, we investigated the contribution of RIPK3/necroptosis to TH-induced photoreceptor degeneration using mice deficient in RIPK3 and the necroptotic mixed lineage kinase domain-like protein (MLKL). Wild-type (C57BL/6) and mutant mice at postnatal day 30 received triiodothyronine (T3, 20 µg/mL in drinking water) for four weeks, followed by the evaluation of photoreceptor survival/death and retinal function. Deletion of *Ripk3* preserved photoreceptor integrity against T3-induced degeneration, evidenced by improved retinal morphology, increased cone density, improved retinal light responses, and reduced cell death. This protection was observed in both global and photoreceptor-specific *Ripk3* knockout mice. In contrast, the deletion of *Mlkl* did not protect photoreceptors. This work supports the view that RIPK3, but not MLKL, contributes to TH-induced photoreceptor degeneration. The lack of protection from *Mlkl* deletion suggests that RIPK3’s action is likely mediated via a necrosome-independent mechanism. These findings provide significant insight into how TH signaling induces photoreceptor degeneration and implicate RIPK3 as a potential therapeutic target.

## 1. Introduction

Rod and cone photoreceptors are vital for vision. Photoreceptors degenerate in a variety of pathological conditions, including inherited retinal degenerative diseases such as retinitis pigmentosa, Leber congenital amaurosis (LCA), and cone-rod dystrophies, and age-related retinal degenerative diseases such as age-related macular degeneration (AMD) and diabetic retinopathy. Inherited retinal degenerative diseases affect approximately 1 in 3000 individuals globally, and AMD is the leading cause of blindness in older adults. Despite the highly heterogeneous nature of these diseases, degenerating photoreceptors often display common cellular issues, including oxidative damage [1,2], inflammatory lesions [3,4,5], apoptosis [6,7], and necroptosis [3,5,8]. Understanding these common cellular features can help us find better ways to manage photoreceptor degeneration and preserve vision.

TH signaling regulates cell proliferation, differentiation, and metabolism across various tissues [9,10,11]. In the retina, TH regulates retinal development and cone opsin expression and is associated with photoreceptor viability. Suppression of TH signaling with an anti-thyroid drug or targeting the intracellular TH components iodothyronine deiodinases and TH receptors has been shown to protect photoreceptors in LCA RPE65-deficient mice [12,13,14,15,16] and in NaIO_3_-induced AMD model mice [17,18]. Conversely, stimulation of TH signaling, either via triiodothyronine (T3) treatment or the deletion of T3-degrading enzymes, induces photoreceptor degeneration. This degeneration manifests as impaired retinal morphology/integrity, reduced retinal function, increased photoreceptor cell stress/death, and increased retinal glial cell activation [14,19,20,21]. Moreover, TH signaling has strong associations with human retinal disease. Population and patient-based studies suggest that elevated TH signaling is associated with an increased incidence of AMD [22,23,24,25,26,27,28,29,30,31]. Optical coherence tomography evaluations have revealed macular thinning in patients with thyroid-associated ophthalmopathy [32,33]. The influence of TH signaling extends beyond the retina with established links to Alzheimer’s disease [34,35] and Parkinson’s disease [36], highlighting its broad impact on neurodegenerative conditions.

Receptor-interacting protein kinase 3 (RIPK3) signaling is a crucial biological process involved in cell death/necroptosis. RIPK3 is a central mediator of necroptosis/programmed necrotic cell death. It works with RIPK1 and mixed lineage kinase domain-like (MLKL) to form the necrosome, leading to the execution of necroptosis, characterized by cell membrane rupture, the release of cellular contents, and cell death [37,38]. In addition to this canonical necroptosis pathway, RIPK3 has been implicated in other cellular processes, including inflammation [39,40], mitochondrial metabolism/bioenergetics [41], and apoptosis [42], though the underlying mechanisms and context-dependency are still being investigated. Our previous observations suggested a potential involvement of RIPK3 and/or necroptotic signaling in TH-induced photoreceptor degeneration, as T3-induced photoreceptor death was accompanied by an increased expression of RIPK3 and MLKL [19]. The current work investigated the role of RIPK3/necroptotic signaling in TH-induced photoreceptor degeneration using *Ripk3*-deficient mice and *Mlkl*-deficient mice. Our findings revealed that the deletion of *Ripk3* significantly preserved photoreceptors against T3-induced degeneration. However, the deletion of *Mlkl* did not result in photoreceptor protection. This work demonstrates the contribution of RIPK3 to TH-induced photoreceptor degeneration. The lack of protection observed with *Mlkl* deletion suggests that the action of RIPK3 is likely mediated via a necrosome-independent or non-canonical mechanism.

## 2. Results

### 2.1. Deletion of Ripk3 Preserved Retinal/Rod Integrity After T3 Treatment

**Figure 1:**
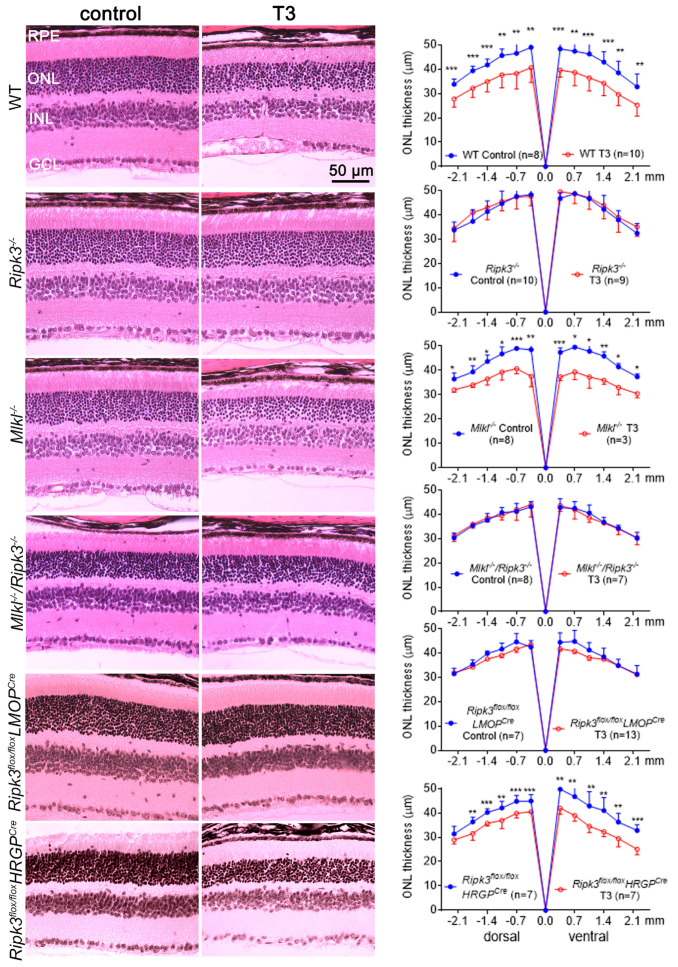
Deletion of *Ripk3* preserved retinal/rod integrity after T3 treatment. C57BL/6, *Ripk3^−/−^*, *Mlkl^−/−^*, *Mlkl^−/−^/Ripk3^−/−^*, rod-specific knockout (*Ripk3^flox/flox^*/*LMOP^Cre^*), and cone-specific knockout (*Ripk3^flox/flox^*/*HRGP^Cre^*) mice at one month of age received T3 treatment for 30 days and were then evaluated for retinal morphology/rod integrity using H&E staining. Shown are representative light microscopic images of H&E-stained retinal cross-sections and the corresponding quantitative analysis. ONL, outer nuclear layer; INL, inner nuclear layer; and GCL, ganglion cell layer. Data are presented as means ± SD for 3–13 mice per group. Unpaired Student’s *t*-test/Mann–Whitney test was used to test for differences between two groups of data (* *p* < 0.05, ** *p* < 0.01, and *** *p* < 0.001, compared with their respective untreated controls).

We examined the effects of *Ripk3* deletion on retinal morphology using *Ripk3^−/−^* mice. The role of MLKL was also examined using *Mlkl^−/−^* and *Mlkl^−/−^/Ripk3^−/−^* mice. After treatment with T3 (20 µg/mL in drinking water) for 30 days, mice were evaluated for retinal morphology/integrity by hematoxylin and eosin (H&E) staining of retinal cross-sections. Our evaluation showed that the deletion of *Ripk3* preserved retinal structural integrity from T3-induced damage/cell loss (Figure 1). T3 treatment significantly impacted the thickness of the outer nuclear layer (ONL) in wild-type (C57BL/6) mice; quantitative analysis showed a significant detriment to the retinal layers in T3-treated mice compared to the controls. Deletion of *Ripk3* completely reversed the damage induced by T3 and provided substantial protection to the retinal layers. There was virtually no difference in ONL thickness between the T3-treated and untreated *Ripk3^−/−^* mice. However, *Mlkl^−/−^* mice did not show any retinal preservation, with the ONL displaying thinning like that seen in wild-type mice after T3 treatment. Meanwhile, *Mlkl^−/−^/Ripk3^−/−^* mice showed a protection similar to that in *Ripk3^−/−^* mice. Since rods constitute over 95% of the total photoreceptor population in a mammalian retina, this protection from ONL thinning primarily reflects rod preservation. To examine the specific effect of *Ripk3* deletion in rods and cones, we utilized rod-specific knockout (*Ripk3^flox/flox^*/*LMOP^Cre^*) and cone-specific knockout (*Ripk3^flox/flox^*/*HRGP^Cre^*) mice. The *Ripk3^flox/flox^*/*LMOP^Cre^* mice showed retinal protection similar to that of *Ripk3^−/−^* mice (Figure 1). In contrast, *Ripk3^flox/flox^*/*HRGP^Cre^* mice treated with T3 had a reduced ONL thickness compared to the untreated controls (Figure 1). This likely reflects the fact that cones represent only 3–5% of the total photoreceptor population in the mouse retina, and the effects of TH on rods still contributed to the overall reduction in ONL thickness.

### 2.2. Deletion of Ripk3 Preserved Cones After T3 Treatment

**Figure 2:**
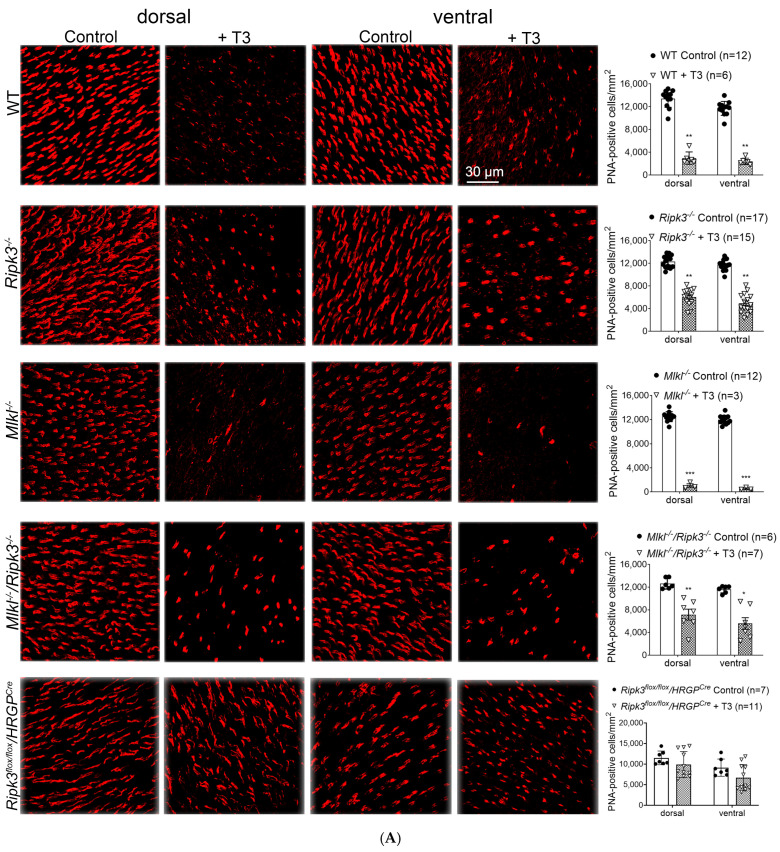

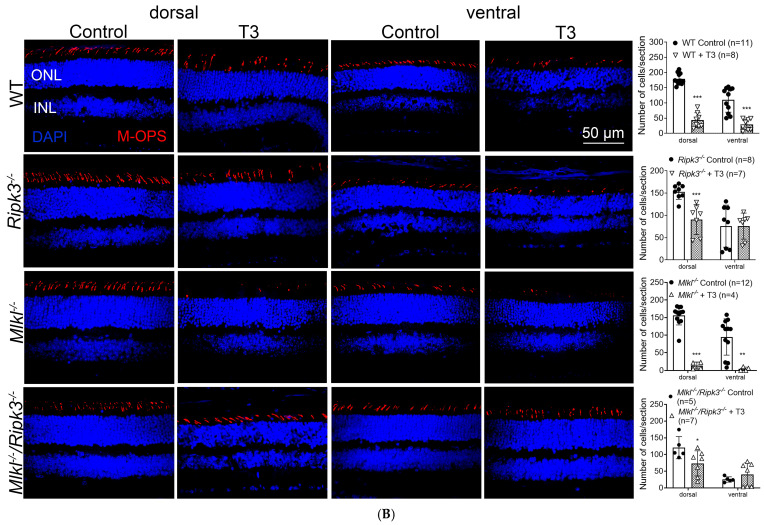
Deletion of *Ripk3* preserved cones after T3 treatment. C57BL/6, *Ripk3^−/−^*, *Mlkl^−/−^*, *Mlkl^−/−^/Ripk3^−/−^*, and *Ripk3* cone-specific knockout (*Ripk3^flox/flox^*/*HRGP^Cre^*) mice at one month of age received T3 treatment (20 µg/mL) for 30 days and were then evaluated for cone density by PNA labeling of retinal whole mounts and M-opsin labeling of retinal cross-sections. (**A**) Shown are representative confocal images of PNA-labeled retinal whole mounts and corresponding quantitative analysis. (**B**) Shown are confocal images of M-opsin labeling of retinal cross-sections and corresponding quantitative analysis. ONL, outer nuclear layer; INL, inner nuclear layer. Data are presented as means ± SD for 3–17 mice per group. Unpaired Student’s *t*-test/Mann–Whitney test was used to test for differences between two groups of data (* *p* < 0.05, ** *p* < 0.01, and *** *p* < 0.001, compared with their respective untreated controls).

We next examined the effects of *Ripk3* deletion on cone preservation. Wild-type, *Ripk3^−/^*^−^, *Mlkl^−/−^*, and *Mlkl^−/−^/Ripk3^−/−^* mice received T3 treatment (20 µg/mL in drinking water) for 30 days and were then evaluated for cone density using PNA immunolabeling on retinal whole mounts and M-opsin immunolabeling on retinal cross-sections. PNA labeling showed that *Ripk3* deletion preserved cones from T3-induced cell loss (Figure 2A). Wild-type mice exhibited a dramatic 79% reduction in PNA-labeled cones after T3 treatment compared to the untreated controls. In contrast, *Ripk3^−/−^* mice showed cone preservation against T3-induced cell loss, with only a 55% reduction in cone density from control levels. However, *Mlkl^−/−^* mice showed no cone preservation; their post-T3 cone density was comparable to or even lower than that of T3-treated wild-type mice (94% reduction). Nevertheless, *Mlkl^−/−^/Ripk3^−/−^* mice showed a protection comparable to *Ripk3^−/−^* mice (48% reduction). To assess cone-specific effects, we employed *Ripk3^flox/flox^*/*HRGP^Cre^* mice, which also exhibited significant protection to *Ripk3^−/−^* mice (21% reduction) (Figure 2A). M-opsin labeling on retinal cross-sections showed similar findings. In wild-type mice, T3 treatment reduced the cone density by approximately 75% compared to the untreated controls. The deletion of *Ripk3* preserved cones against T3-induced cell loss, with only a 27% reduction in cone density relative to the untreated controls (Figure 2B). *Mlkl^−/−^* mice did not show any cone preservation, with their post-treatment cone density being similar to wild-type mice treated with T3 (93% reduction), while *Mlkl^−/−^/Ripk3^−/−^* mice exhibited a protection comparable to *Ripk3^−/−^* mice (39% reduction in the dorsal area and no reduction in the ventral area) (Figure 2B).

### 2.3. Deletion of Ripk3 Preserved Retinal Function After T3 Treatment

**Figure 3:**
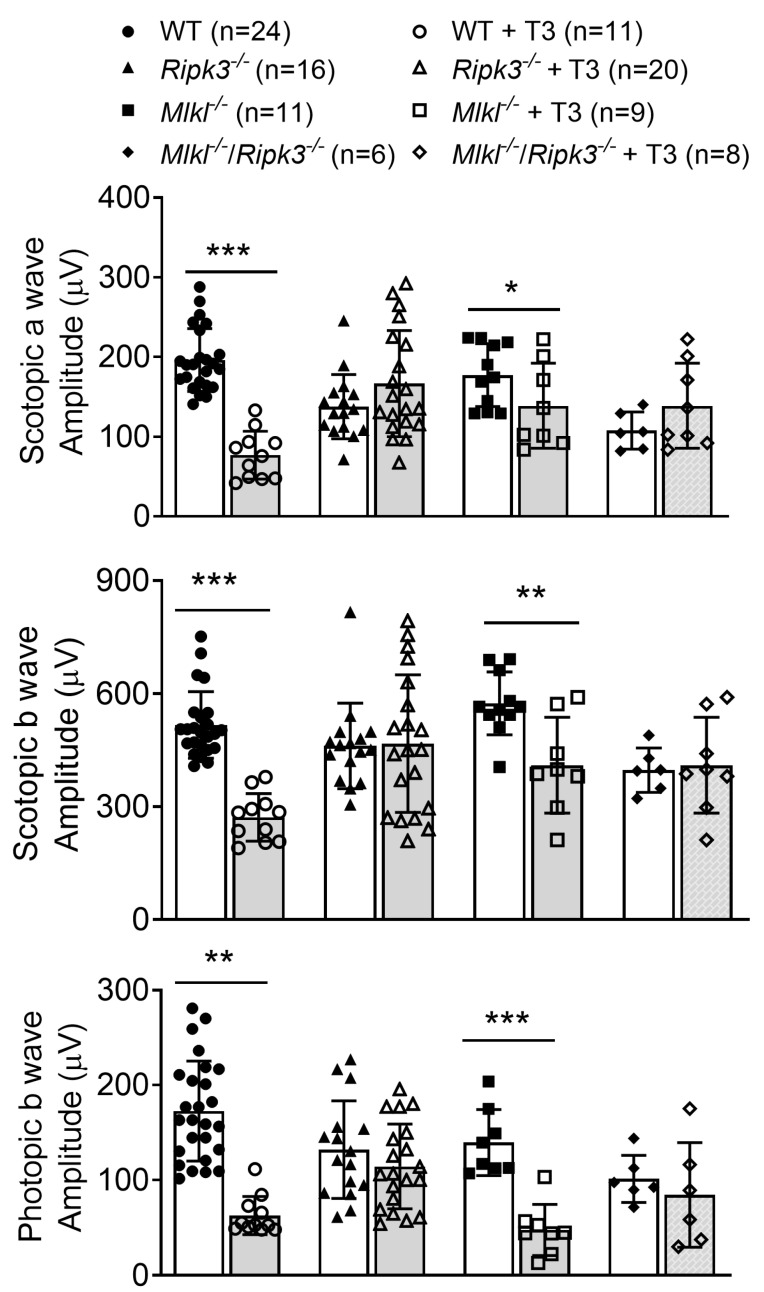
Deletion of *Ripk3* preserved retinal function after T3 treatment. C57BL/6, *Ripk3^−/−^*, *Mlkl^−/−^*, and *Mlkl^−/−^/Ripk3^−/−^* mice received T3 treatment (20 µg/mL in drinking water) for 30 days and were then evaluated for light responses using ERG recordings. Shown are the results of scotopic and photopic light response amplitudes of T3-treated mice compared with untreated controls. Data are represented as means ± SD for 6–24 mice per group. Unpaired Student’s *t*-test/Mann–Whitney test was used to test for differences between two groups of data (* *p* < 0.05, ** *p* < 0.01, and *** *p* < 0.001, compared with their respective untreated controls).

The effects of *Ripk3* deletion on retinal function after T3 treatment were evaluated using electroretinography (ERG) recordings. Wild-type, *Ripk3^−/−^*, *Mlkl^−/−^*, and *Mlkl^−/−^*/*Ripk3^−/−^* mice received T3 treatment (20 µg/mL in drinking water) for 30 days and were then assessed for scotopic (rod function) and photopic (cone function) light response. ERG analysis showed that deletion of *Ripk3* preserved photoreceptors from T3-induced impairment (Figure 3). Wild-type mice treated with T3 had significantly impaired light responses, with the scotopic a-wave, scotopic b-wave, and photopic b-wave reduced by about 61%, 48%, and 64% of the untreated control levels, respectively. In contrast, *Ripk3^−/−^* mice maintained near-normal ERG responses after T3 treatment, showing no significant reduction in either scotopic or photopic responses. *Mlkl^−/−^* mice showed some scotopic rescue, with the scotopic a-wave and scotopic b-wave reduced by approximately 22% and 29% of untreated control levels, respectively, but showed no photopic rescue (66% reduction). *Mlkl^−/−^*/*Ripk3^−/−^* mice showed a light response rescue comparable to that observed in *Ripk3^−/−^* mice with no reduction in ERG responses.

### 2.4. Deletion of Ripk3 Reduced Photoreceptor Apoptosis After T3 Treatment

**Figure 4:**
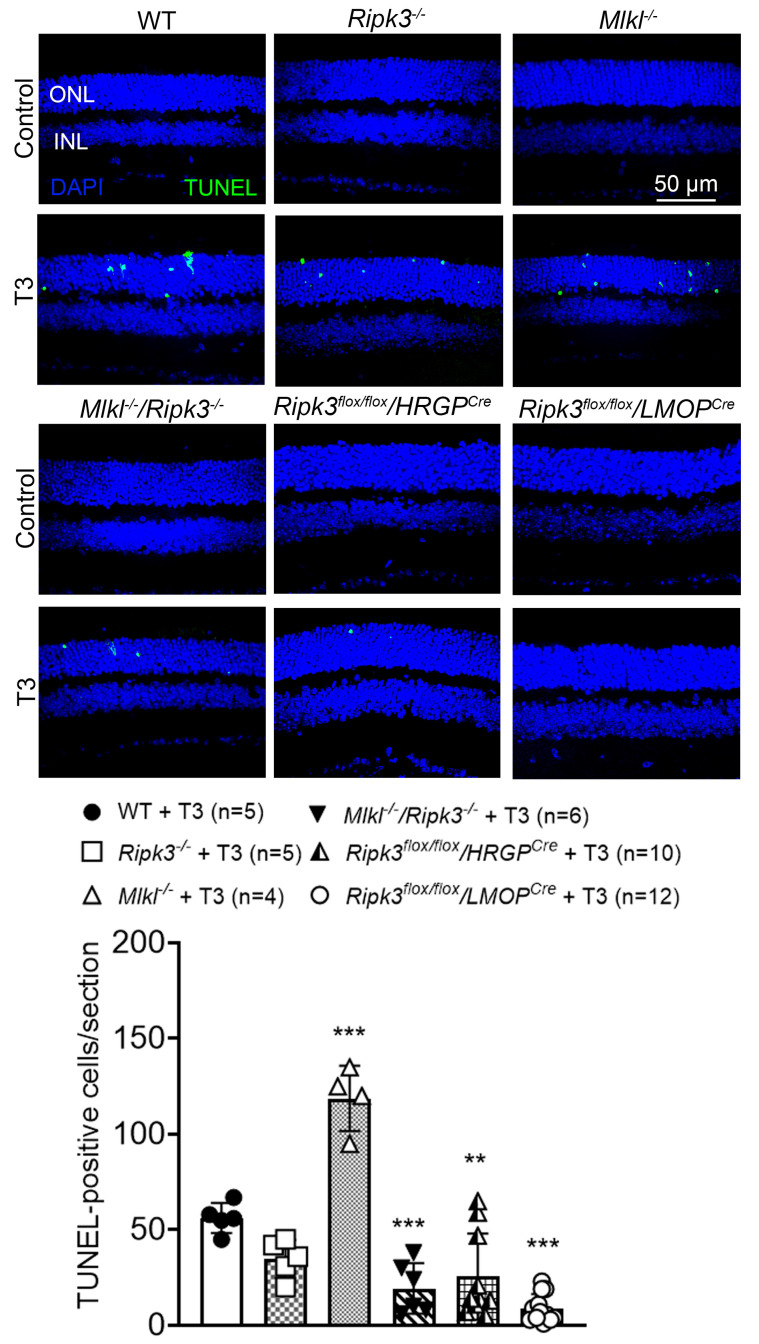
Deletion of *Ripk3* reduced photoreceptor apoptosis after T3 treatment. C57BL/6, *Ripk3^−/−^*, *Mlkl^−/−^*, *Mlkl^−/−^/Ripk3^−/−^*, *Ripk3* rod-specific knockout (*Ripk3^flox/flox^*/*LMOP^Cre^*), and *Ripk3* cone-specific knockout (*Ripk3^flox/flox^*/*HRGP^Cre^*) mice at one month of age received T3 treatment (20 µg/mL) for 30 days and were then evaluated for cell death. Shown are confocal images of TUNEL labeling on retinal cross-sections and corresponding quantitative analysis. ONL, outer nuclear layer; INL, inner nuclear layer. Data are presented as means ± SD for 3–7 mice per group. One-way ANOVA was used for significance within sets of data, followed by Dunnett’s multiple comparisons test (** *p* < 0.01, and *** *p* < 0.001, compared with wild-type mice after T3 treatment).

Photoreceptor cell death/apoptosis after T3 treatment was evaluated by TUNEL labeling on retinal cross-sections. C57BL/6, *Ripk3^−/−^*, *Mlkl^−/−^*, *Mlkl^−/−^*/*Ripk3^−/−^*, *Ripk3* rod-specific knockout (*Ripk3^flox/flox^*/*LMOP^Cre^*), and *Ripk3* cone-specific knockout (*Ripk3^flox/flox^*/*HRGP^Cre^*) mice received the T3 treatment (20 µg/mL in drinking water) for 30 days and were then evaluated for photoreceptor cell apoptosis. TUNEL labeling showed that the deletion of *Ripk3* reduced T3-induced photoreceptor apoptosis (Figure 4). There was almost no detection of TUNEL-positive cells in untreated mice of all genotypes. Treatment with T3 induced cell death in all groups, shown as increased numbers of TUNEL-positive cells on retinal cross-sections. Quantitative analysis revealed that *Ripk3* deletion attenuated this increase in apoptosis (Figure 4). The number of TUNEL-positive cells was reduced in *Ripk3*^−/−^ mice (38% reduction), though not to a statistically significant degree. The number of TUNEL-positive cells was significantly reduced in the *Mlkl^−/−^/Ripk3^−/−^* (66% reduction), *Ripk3* cone-specific knockout (55% reduction), and *Ripk3* rod-specific knockout (84% reduction), compared to that in wild-type mice. However, *Mlkl^−/−^* mice did not show any protection and, in fact, showed a significant increase in the number of TUNEL-positive cells relative to wild-type mice (111% increase) (Figure 4).

### 2.5. Deletion of Ripk3 Reduced Photoreceptor DNA Damage/Oxidative Stress After T3 Treatment

**Figure 5:**
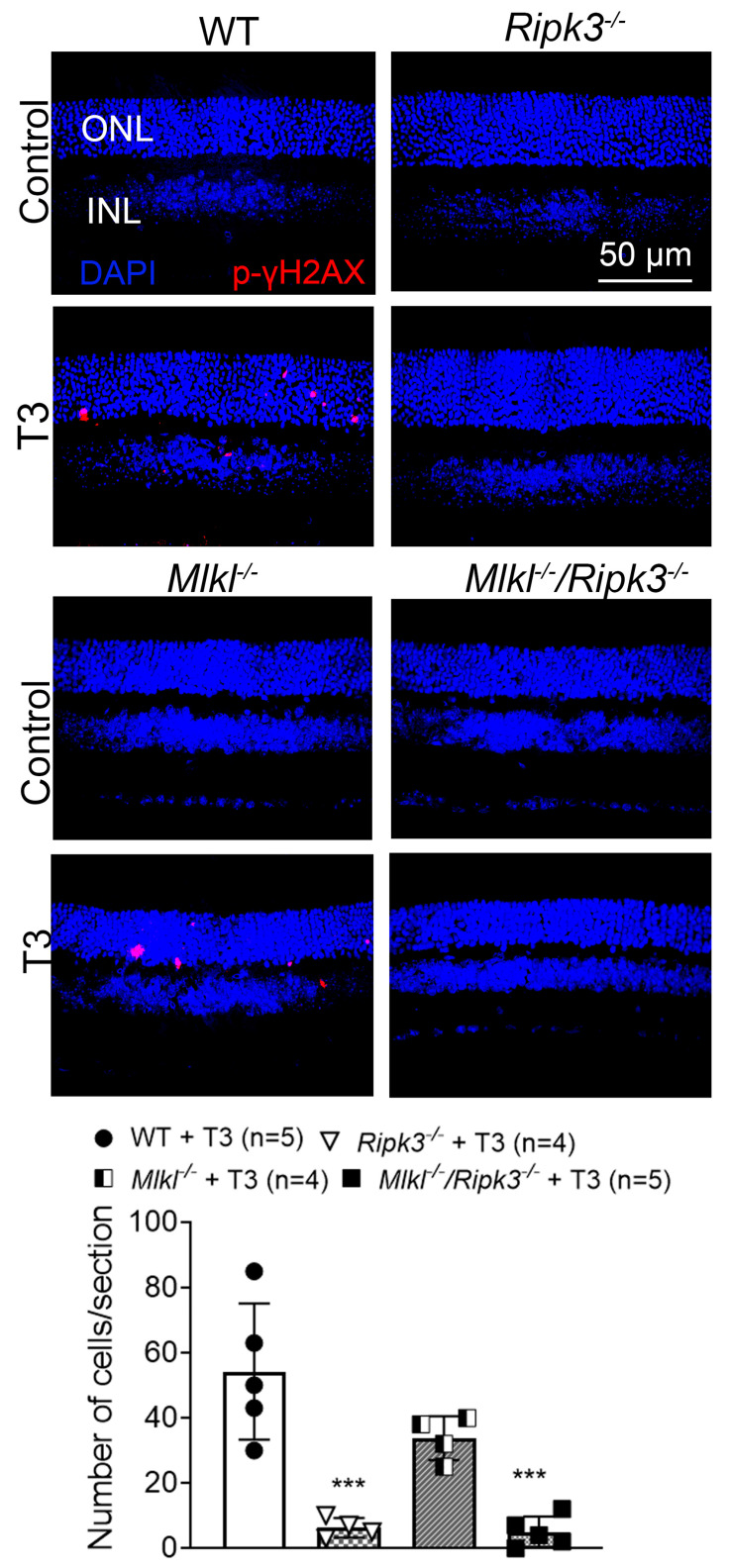
Deletion of *Ripk3* reduced photoreceptor oxidative stress after T3 treatment. C57BL/6, *Ripk3^−/−^*, *Mlkl^−/−^*, and *Mlkl^−/−^/Ripk3^−/−^* mice received T3 treatment (20 µg/mL in drinking water) for 30 days and were then evaluated for oxidative stress/damage in the retina using p-γH2AX labeling. Shown are representative confocal images of p-γH2AX labeling on retinal cross-sections of T3-treated mice compared with untreated controls and corresponding quantitative analysis. ONL, outer nuclear layer; INL, inner nuclear layer. Data are represented as means ± SD for 4–5 mice per group. One-way ANOVA was used for significance within the sets of data, followed by Dunnett’s multiple comparisons test (*** *p* < 0.001, compared with wild-type mice after T3 treatment).

We further investigated the effect of *Ripk3* deletion on DNA damage/oxidative stress in the retina after T3 treatment. C57BL/6, *Ripk3^−/−^*, *Mlkl^−/−^*, and *Mlkl^−/−^*/*Ripk3^−/−^* mice received T3 treatment (20 µg/mL in drinking water) for 30 days and were then evaluated for oxidative stress/damage by p-γH2AX labeling on retinal cross-sections. Phosphorylated γH2AX (p-γH2AX) is a widely used and highly sensitive biomarker for DNA double-strand breaks. As oxidative stress can be a cause of these DNA double-strand breaks, this labeling was used as an indirect marker for assessing oxidative stress in the retina. Our examinations showed that the deletion of *Ripk3* reduced T3-induced photoreceptor oxidative stress (Figure 5). There was nearly no detection of p-γH2AX-labeled cells in untreated mice of all genotypes. Treatment with T3 increased the number of p-γH2AX-positive cells in wild-type mice. The global deletion of *Ripk3* significantly reversed this increase, with a substantially lower number of p-γH2AX-positive cells compared to wild-type mice (88% reduction). However, *Mlkl^−/−^* mice did not show significant protection compared to that in T3-treated wild-type mice (38% reduction). *Mlkl^−/−^/Ripk3^−/−^* mice exhibited a significantly reduced number of p-γH2AX-positive cells, comparable to that observed in *Ripk3^−/−^* mice (91% reduction) (Figure 5).

### 2.6. Deletion of Ripk3 Reduced Retinal Macroglial Cell Activation After T3 Treatment

**Figure 6:**
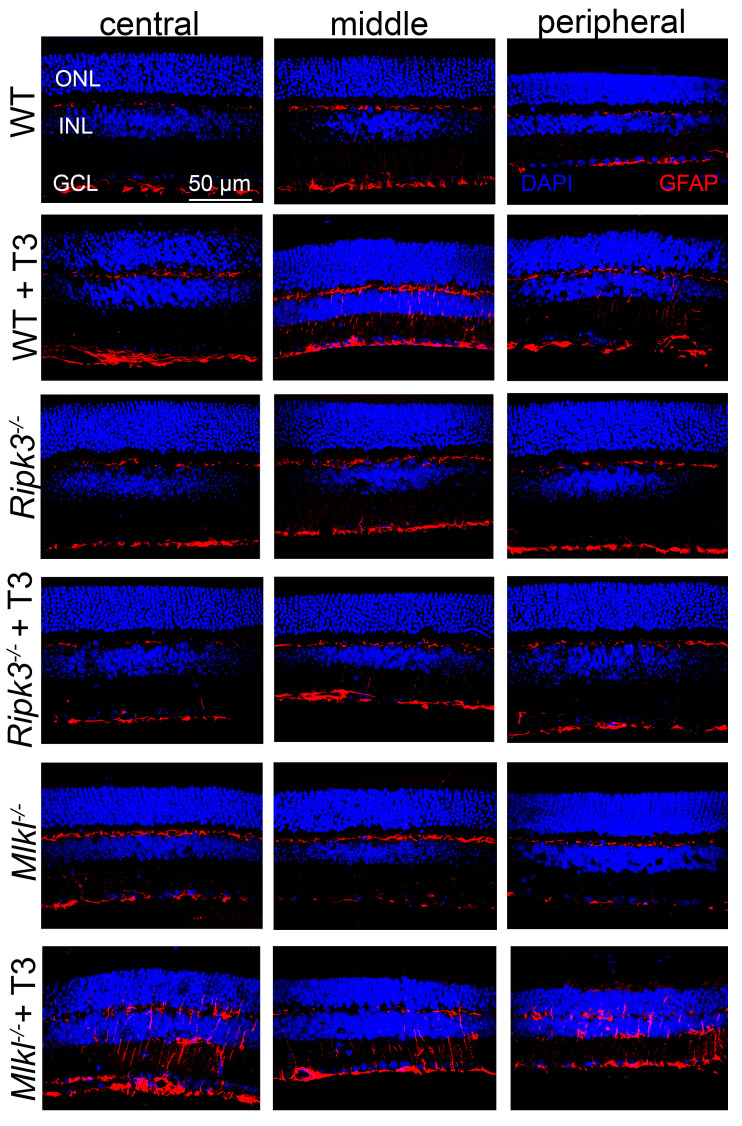
Deletion of *Ripk3* reduced retinal macroglial cell activation after T3 treatment. C57BL/6, *Ripk3^−/−^*, and *Mlkl^−/−^* mice received T3 treatment (20 µg/mL in drinking water) for 30 days and were then evaluated for activation of Müller glial cells in the retina. Shown are representative confocal images of GFAP labeling on retinal cross-sections of T3-treated mice compared with their respective untreated controls. ONL, outer nuclear layer; INL, inner nuclear layer; and GCL, ganglion cell layer.

We also investigated the effect of *Ripk3* deletion on retinal macroglial cell (Müller cell and astrocyte) activation after the T3 treatment. C57BL/6, *Ripk3^−/−^*, and *Mlkl^−/−^* mice received T3 treatment (20 µg/mL in drinking water) for 30 days and were evaluated for the activation of macroglial cells using GFAP labeling on retinal cross-sections. Our immunolabeling showed that the global deletion of *Ripk3* reduced T3-induced retinal macroglial cell activation (Figure 6). GFAP labeling was increased in wild-type mice treated with T3 compared to the untreated controls, indicating robust glial activation. This increase was notably diminished in *Ripk3^−/−^* mice. However, GFAP immunolabeling in *Mlkl^−/−^* mice after T3 treatment was at a comparable level to, or even higher than, the levels observed in wild-type mice.

### 2.7. Deletion of Ripk3 Diminished the Expression of the Inflammatory Genes After T3 Treatment

**Figure 7:**
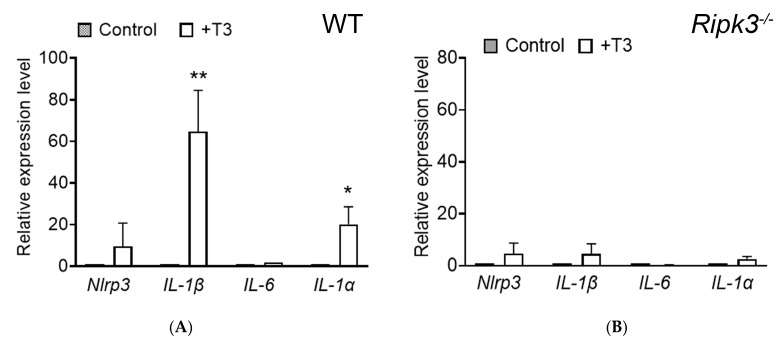
Deletion of *Ripk3* diminished expression of the inflammatory genes after T3 treatment. C57BL/6 and *Ripk3^−/−^* mice received T3 treatment (20 µg/mL in drinking water) for 30 days and were then evaluated for expression of the genes involved in inflammatory response. Shown are qRT-PCR results for expression levels of the inflammatory genes in the retina prepared from wild-type (**A**) and *Ripk3^−/−^* (**B**) mice. Data are represented as means ± SD of 3–5 assays using retinas prepared from 3 to 9 mice per group. Unpaired Student’s *t*-test/Mann–Whitney test was used to test for differences between two groups of data (* *p* < 0.05, ** *p* < 0.01, compared with their respective untreated controls).

We also examined the effect of *Ripk3* deletion and T3 treatment on the expression of inflammatory genes that are sometimes associated with RIPK3 activity. C57BL/6 and *Ripk3^−/−^* mice received T3 treatment (20 µg/mL in drinking water) for 30 days and were then evaluated for expression of the inflammatory genes *Nlrp3*, *Il-1α*, *Il-1β*, and *Il-6* using qRT-PCR. Our assays showed that global deletion of *Ripk3* mitigated the expression of the inflammatory genes induced by T3 (Figure 7). In wild-type mice, T3 treatment led to approximately 20-fold and 65-fold increases in *Il-1α* and *Il-1β* expression, respectively. These elevations were nearly completely abolished in *Ripk3^−/−^* mice (87% and 93% reduction, respectively), indicating a strong anti-inflammatory effect of *Ripk3* deletion in the context of T3-induced retinal stress.

## 3. Discussion

### 3.1. RIPK3 Contributes to T3-Induced Photoreceptor Degeneration, and This Action Is Likely via a Necrosome-Independent Mechanism

The present work investigated the role of RIPK3 and MLKL in TH signaling-induced retinal/photoreceptor degeneration in mice. Deletion of *Ripk3* preserved retinal morphology and function against T3-induced damage and reduced photoreceptor cell death, oxidative stress/damage, and retinal macroglial cell activation. These observations were obtained in *Ripk3* global knockout [43] and *Ripk3* photoreceptor-specific knockout mice, demonstrating the role of RIPK3 in the T3-induced degeneration of photoreceptors. The protection against T3 treatment was observed in both rods and cones, suggesting an equivalent role of RIPK3 in T3-induced damage to these photoreceptors. In contrast, the global deletion of *Mlkl* did not render any protection against T3 treatment [43]. *Mlkl^−/−^* mice showed a similar level of retinal damage in response to the T3 treatment as was seen in the wild-type mice. Furthermore, the simultaneous deletion of both *Ripk3* and *Mlkl* showed levels of protection similar to those seen in the single knockout *Ripk3^−/−^* mice [43], suggesting that the protection seen in *Mlkl^−/−^*/*Ripk3^−/−^* mice is most likely due to the deletion of *Ripk3* alone. MLKL is the well-established terminal effector of the necroptosis pathway. Cell membrane disruption in necroptosis is achieved by MLKL after its phosphorylation and oligomerization [44,45,46], and MLKL deficiency/knockout abolishes the function of the necrosome/necroptosis [47,48]. Thus, the lack of protection in *Mlkl^−/−^* mice supports the view that the contribution of RIPK3 to TH-induced photoreceptor degeneration is likely mediated via a necrosome-independent/non-canonical mechanism. These new data support and expand upon previous evidence that RIPK3 contributes to retinal degeneration. The genetic deletion of *Ripk3* was shown to protect photoreceptors in mouse models of retinal degeneration, such as *rd10* mice [49], retinal detachment [50], and alkylation-induced degeneration [51].

It was noted that the global *Ripk3* knockout mice did not protect photoreceptors from T3 as much as the cone-specific *Ripk3* knockout mice (see Figure 2A). This is an interesting finding, though we do not have a full explanation for it yet. It appears that the RIPK3 deficiency in other retinal cell types might have negatively impacted the photoreceptors.

It should be pointed out that the knockout mouse lines under untreated conditions show some retinal degeneration. This includes a reduced ONL thickness in *Mlkl^−/−^*/*Ripk3^−/−^* and *Ripk3^flox/flox^*/*LMOP^Cre^* mice (see Supplementary Figure S1), reduced cone density in *Mlkl^−/−^*/*Ripk3^−/−^* mice (see Supplementary Figure S2), reduced rod ERG response in *Ripk3^−/−^* and *Mlkl^−/−^*/*Ripk3^−/−^* mice (see Supplementary Figure S3A), and reduce cone ERG response in *Ripk3^−/−^*, *Mlkl^−/−^*, and *Mlkl^−/−^*/*Ripk3^−/−^* mice (see Supplementary Figure S3B). The cone density was evaluated using PNA labeling on retinal whole mounts and M-opsin labeling on retinal cross-sections. A reduction in cone density in the double knockout mice without T3 treatment was observed in the retinal cross-section analysis but not in the whole mount analysis (see Supplementary Figure S2). This discrepancy is likely due to the different methodologies and molecular markers used. Tissue section assays generally offer a higher resolution and precision, and M-opsin labeling is likely more specific than PNA labeling. Nevertheless, the retinal phenotype in RIPK3-deficient mice and MLKL-deficient mice suggests a role of these proteins in retinal/photoreceptor maintenance, warranting further investigation.

### 3.2. RIPK3 Plays a Role in T3-Induced Photoreceptor Apoptosis

Although RIPK3 is a key protein in the classical necroptotic (necrosome) pathway, accumulating evidence indicates that it also has functions independent of necroptosis, including promoting apoptosis under certain conditions [42]. In this study, we show that deletion of *Ripk3* significantly reduced the T3-induced photoreceptor apoptosis in both *Ripk3^−/−^* and *Mlkl^−/−^/Ripk3^−/−^* mice, supporting the contribution of RIPK3 to T3-induced photoreceptor apoptosis. Nevertheless, the precise mechanism by which RIPK3 promotes apoptosis is not fully understood. Previous studies have shown that RIPK3 can act as a scaffold to recruit other proteins like FADD (Fas-associated death domain) and caspase-8 to form a complex that activates caspase-8 and triggers apoptosis [42,52]. It has also been suggested that the decision/balance between apoptosis and necroptosis can be dictated by the availability of MLKL. If MLKL is absent, RIPK3 activation can lead to apoptosis [53]. Consistent with this model, we observed that *Mlkl^−/−^* mice exhibited increased photoreceptor apoptosis following T3 treatment, with the number of TUNEL-positive cells doubling compared to the wild-type controls (see Figure 4). Altogether, the findings from *Ripk3^−/−^*, *Mlkl^−/−^/Ripk3^−/−^*, and *Mlkl^−/−^* mice show that RIPK3 contributes to T3-induced photoreceptor apoptosis. Nevertheless, how RIPK3 facilitates apoptosis of photoreceptors after T3 treatment is unclear at this time and merits further investigation.

### 3.3. RIPK3 Plays a Role in T3-Induced Photoreceptor DNA Damage/Oxidative Stress

In this study, we show that the deletion of *Ripk3* greatly reduced T3-induced photoreceptor DNA damage/oxidative stress in both *Ripk3^−/−^* and *Mlkl^−/−^*/*Ripk3^−/−^* mice. The number of p-γH2AX-labeled cells in the mutant mice after T3 treatment was about 5–10% of the level in the wild-type mice (see Figure 5). These findings support the contribution of RIPK3 to T3-induced DNA damage/oxidative stress. It has been suggested that RIPK3 interplays significantly with oxidative stress. Oxidative stress/hydrogen peroxide (H_2_O_2_) can activate RIPK3 [54], which in turn can further promote oxidative stress, likely by increasing aerobic respiration, upregulating NADPH oxidase-4 (NOX4), and inhibiting mitochondrial complexes I and III [55,56]. T3 induces photoreceptor oxidative stress/damage and impairs oxidative phosphorylation in the retina [19,21,57]. T3 also induces the expression of RIPK3 in the retina [19], likely through the direct action of T3 and/or the indirect action of T3-induced oxidative stress. The increased expression/function of RIPK3 may promote further oxidative stress/damage through the feedback mechanisms cited above, as evidenced by our observation that the deletion of *Ripk3* alleviates T3-induced oxidative stress/damage in the retina. Importantly, T3-induced oxidative stress/damage alone appears to be sufficient to cause photoreceptor death, as treatment with antioxidants has been shown to effectively reduce retinal degeneration after T3 treatment [19,21].

### 3.4. RIPK3 Plays a Role in T3-Induced Upregulation of the Inflammatory Genes in the Retina and Activation of Retinal Macroglial Cells

Excessive TH signaling induces the expression of genes involved in inflammatory responses [19,21]. In our study, treatment with T3 induced the expression of Il-1α and Il-1β in wild-type mice, but their upregulation was completely abolished in Ripk3^−/−^ mice. Among the necrosome-independent actions of RIPK3, its role in inflammation has been best characterized. RIPK3 can induce the expression of inflammatory cytokines, including IL-1β and chemokines, primarily through its action on the inflammasome [39,58]. This upregulation may also involve the NF-κB (nuclear factor kappa-light-chain-enhancer of activated B cells) signaling pathway, a master regulator of pro-inflammatory gene expression [59,60]. Nevertheless, there is a strong and well-documented connection between inflammation and apoptosis in the retina. In many retinal diseases, inflammation plays a significant role in triggering and exacerbating the apoptotic cell death of retinal cells/photoreceptors [61].

A well-established connection exists between macroglial cell activation and inflammation in the retina. While this activation can offer early protection and a proper stress response, it often leads to retinal cell stress and death [62,63]. Activation of Müller cells and astrocytes, as indicated by increased expression of GFAP, has been shown in a variety of animal models of retinal degeneration, including TH-induced retinal degeneration [19,21]. Our recent transcriptomic studies showed that there were approximately 180 differentially expressed genes (DEGs) in Müller glial cells and approximately 160 DEGs in astrocytes after T3 treatment [57], highlighting significant transcriptional responses in retinal macroglia. Our current study shows that GFAP immunolabeling after T3 treatment in *Ripk3^−/−^* mice was reduced, compared to that in the wild-type mice and *Mlkl^−/−^* mice, supporting the role of RIPK3 in the activation of retinal macroglial cells under T3-induced stress conditions. It is likely that the activation of the macroglial cells in response to T3 treatment results from both photoreceptor stress/degeneration and the direct action of T3 on these cells. It is also reasonable to presume that the observed reduction in GFAP labeling/macroglial cell activation in *Ripk3^−/−^* mice may result from both reduced photoreceptor stress/degeneration and the reduced stress responses of macroglial cells themselves.

### 3.5. Potential Influence of RPE in TH-Induced Photoreceptor Degeneration

Photoreceptors depend on the retinal pigment epithelium (RPE) for maintaining their normal function and integrity. TH signaling affects RPE integrity. TH receptors are expressed in the RPE [18,64,65], and excessive TH signaling is known to be harmful to the RPE [66], yet the underlying mechanism remains unclear. Additionally, TH signaling contributes to RPE damage after oxidative stress [17,18]. These observations suggest that TH signaling-induced photoreceptor degeneration may be, at least partially, mediated by RPE damage. Nevertheless, the RPE’s influence on TH-induced photoreceptor degeneration warrants a separate investigation.

In summary, the present study demonstrates a critical role of RIPK3 in T3-induced photoreceptor degeneration. Deletion of *Ripk3* preserved photoreceptors against T3-induced degeneration and stress responses and preserved retinal function. The lack of protection from *Mlkl* deletion suggests that the action of RIPK3 in T3-induced photoreceptor degeneration is likely mediated via a necrosome-independent/non-canonical mechanism. The findings provide significant insight into the mechanism by which TH signaling induces photoreceptor degeneration and implicate RIPK3 as a potential therapeutic target.

## 4. Materials and Methods

### 4.1. Mice, Antibodies, and Reagents

Wild-type (C57BL/6J) and *Ripk3^−/−^* lines were obtained from The Jackson Laboratory (Bar Harbor, Maine). The *Mlkl^−/−^* line [67] was provided by Dr. Warren Alexander (The Walter and Eliza Hall Institute of Medical Research, Parkville, VIC, Australia). The *Ripk3^flox/flox^* [68], *LMOP^Cre^* [69], and *HRGP^Cre^* [70] lines were generated as described previously. The *Mlkl^−/−^/Ripk3^−/−^*, *Ripk3^flox/flox^*/*LMOP^Cre^*, and *Ripk3^flox/flox^*/*HRGP^Cre^* lines were generated by crossbreeding. Mice were maintained under cyclic-light (12 h light–dark) conditions. Cage illumination was a 7-foot candle during the light cycle. All animal maintenance and experiments were approved by the local Institutional Animal Care and Use Committee (University of Oklahoma Health Sciences) and conformed to the Guidelines on the Care and Use of Animals of the Society for Neuroscience and the Association for Research in Vision and Ophthalmology. Mice of either sex were used in the experiments and randomly assigned within a litter for the drug treatment or vehicle/untreated experiments. Antibodies and reagents used in the experiments are listed in Table 1.

### 4.2. T3 Treatment

Triiodothyronine (T3) for drinking water was prepared as described [71]. Ten milligrams of T3 (T2877, Sigma-Aldrich, St. Louis, MO, USA) was dissolved in 1.0 mL of 1.0 N NaOH, followed by dilution with tap water for final working concentrations.

### 4.3. Scotopic and Photopic Electroretinography Recordings

Full-field electroretinography (ERG) recordings were conducted as described previously [19]. Briefly, after overnight dark adaptation, mice were anesthetized by intraperitoneal injection of 85 mg/kg ketamine and 14 mg/kg xylazine. ERGs were recorded using an Espion visual electrophysiology system with a Ganzfeld ColorDome system (Diagnosys, Lowell, MA, USA). Potentials were recorded using a gold wire electrode to contact the corneal surface through a layer of 2.5% hypromellose (Gonak, Akorn, Lake Forest, IL, USA). For assessment of scotopic responses, a stimulus intensity of 1.89 log cd·s m^−2^ was presented to dark-adapted dilated mouse eyes. To evaluate photopic responses, mice were adapted to a 1.48 log cd·s m^−2^ light for 7 min, and then a light intensity of 1.89 log cd·s m^−2^ was given. Responses were differentially amplified, averaged, and analyzed using Espion 100 software (Diagnosys).

### 4.4. Eye Preparation, Immunofluorescence Labeling, Confocal Microscopy, and Morphometric Analysis

Retinal whole mounts or cross-sections were prepared for immunofluorescence labeling, as described previously [14]. For retinal whole mount preparations, eyes were enucleated, marked at the superior pole with a green dye, and fixed in 4% paraformaldehyde (PFA) (Polysciences, Warrington, PA, USA) for 1 h at room temperature, followed by removal of the cornea and lens. The eyes were then fixed in 4% PFA for 4–6 h at room temperature, retinas were isolated, and the superior portion was marked for orientation with a small cut. For retinal cross-sections, mouse eyes were enucleated (the superior portion of the cornea was marked with green dye before enucleation) and fixed in Prefer (Anatech Ltd., Battle Creek, MI, USA) for 4 h at room temperature before being transferred into 70% ethanol. Paraffin sections (5 µm thickness) passing vertically through the retina (along the vertical meridian passing through the optic nerve head) were prepared using a Leica microtome (Leica Biosystems, Deer Park, IL, USA).

Immunofluorescence labeling was performed as described previously [14]. Briefly, retinal whole mounts were blocked with Hanks’ balanced salt solution containing 5% BSA and 0.5% Triton X-100 overnight at 4 °C. Peanut-agglutinin (PNA) immunohistochemistry was performed using biotinylated PNA and then streptavidin-Cy3 at room temperature for 1 h. For immunofluorescence staining on sections, after the de-paraffin and rehydration steps, antigen retrieval was performed in 10 mM sodium citrate buffer, pH 6.0, in either a 70 °C (normal) or 97 °C (harsh) water bath. Primary antibody incubation was performed overnight at 4 °C. Slides were mounted and cover-slipped after fluorescence-conjugated secondary antibody incubation and wash steps. Immunofluorescence was imaged using an Olympus FV1000 confocal laser scanning microscope and FluoView imaging software version 4.2b (Olympus, Tokyo, Japan). For retinal morphometric analysis, retinal cross-sections stained with hematoxylin and eosin (H&E) were used to evaluate outer nuclear layer (ONL) thickness, as described previously [14].

### 4.5. TUNEL

Terminal deoxynucleotidyltransferase dUTP nick-end labeling (TUNEL) was performed on paraffin-embedded retinal cross-sections, using an in-situ cell death fluorescein detection kit (Sigma-Aldrich, Catalog#: 11684795910), as described previously. Immunofluorescence signals were imaged using an Olympus FV1000 confocal laser scanning microscope. TUNEL-positive cells in the outer nuclear layer passing through the optic nerve were counted and averaged from at least three sections per eye, from 4 to 12 mice per condition.

### 4.6. RNA Isolation and Quantitative Real-Time PCR

Total RNA preparation and reverse transcription were performed as described previously [17,18]. Briefly, retinas were lysed, and RNA was isolated using a PureLink^TM^ RNA kit (Thermo Fisher Scientific) per manufacturer’s instructions. cDNA was prepared using iScript Reverse Transcription Supermix (Bio-Rad) and was amplified using iTaq Universal SYBR^®^ Green Supermix (Bio-Rad). The primer sets are listed in Table 2. The gene encoding murine hypoxanthine guanine phosphoribosyl transferase 1 (*Hprt1*) was included as an internal control. Quantitative real-time PCR (qRT-PCR) assays were performed using a CFX connected Real-Time PCR Detection System (iCycler, Bio-Rad Laboratories, Hercules, CA, USA). All assessed genes were run in triplicate, and the relative gene expression was calculated based on the ΔΔCt method with conditions normalized to *Hprt1.*

### 4.7. Statistical Analysis

The results are expressed as means  ±  SD of the number of mice. Power analysis was performed to choose the sample size. The analysis indicated that a sample size of 3–5 mice/group for evaluations of retinal degeneration in the mouse retinas would provide at least 80% power (1-β) for a two-sided, two-sample *t*-test at a 0.05 alpha level. One-way ANOVA was used for significance within sets of data, followed by Dunnett’s multiple comparisons test. Unpaired Student’s *t*-test/Mann–Whitney test was used to test for differences between two groups of data. Data were analyzed using non-parametric tests. Differences were considered statistically significant when *p*  <  0.05. Data were analyzed and graphed using GraphPad Prism^®^ software version 10.4.2 (GraphPad Software, San Diego, CA, USA).

## Figures and Tables

**Table 1:** Antibodies/reagents and conditions used in this study.

Antibodies/Reagent	Vendor	Catalog Number	Dilutions Used in IF or IB
3,3′,5-triiodo-L-thyronine	Millipore Sigma (Darmstadt, Germany)	T2877	
DAPI (4,6-Diamidino-2-phenylindole)	Millipore Sigma	D9542	1:2000 (IF)
biotinylated PNA	Vector Labs (Newark, CA, USA)	B-1075	1:200 (IF)
Anti-M-opsin	Millipore Sigma	AB5405	1:200 (IF)
TUNEL	Sigma-Aldrich (Saint Louis, MO, USA)	11684795910	1:10 (IF)
anti-γH2AX (p Ser139)	Novus (Centennial, CO, USA)	NB100-2280	1:200 (IF)
anti-GFAP	DAKO (Glostrup, Denmark)	Z0334	1:500 (IF)
Alexa Fluor^®^ 555 goat anti-rabbit IgG	ThermoFisher Scientific (Waltham, MA USA)	A21428	1:500 (IF)
Streptavidin-Cy3	ThermoFisher Scientific	SA1010	1:500 (IF)

**Table 2:** Primers used in this study.

Gene	Forward Primer	Reverse Primer
*Hprt1*	GCAAACTTTGCTTTCCCTGGTT	CAAGGGCATATCCAACAACA
*Nlrp3*	CTCCAACCATTCTCTGACCAG	ACAGATTGAAGTAAGGCCGG
*Il1α*	TGCAGTCCATAACCCATGATC	ACAAACTTCTGCCTGACGAG
*Il1β*	ACGGACCCCAAAAGATGAAG	TTCTCCACAGCCACAATGAG
*Il6*	CAAAGCCAGAGTCCTTCAGAG	GTCCTTAGCCACTCCTTCTG

## Data Availability

The data generated and analyzed during the current study are available from the corresponding author on reasonable request.

## Supplementary Materials

The supporting information can be downloaded at: https://www.mdpi.com/article/10.3390/ijms26178154/s1.

## References

[1] Shen J., Yang X., Dong A., Petters R.M., Peng Y., Wong F., Campochiaro P.A. (2005). Oxidative damage is a potential cause of cone cell death in retinitis pigmentosa. J. Cell. Physiol..

[2] Komeima K., Rogers B.S., Lu L., Campochiaro P.A. (2006). Antioxidants reduce cone cell death in a model of retinitis pigmentosa. Proc. Natl. Acad. Sci. USA.

[3] Allocca M., Corrigan J.J., Mazumder A., Fake K.R., Samson L.D. (2019). Inflammation, necrosis, and the kinase RIP3 are key mediators of AAG-dependent alkylation-induced retinal degeneration. Sci. Signal..

[4] Cruz-Guilloty F., Saeed A.M., Echegaray J.J., Duffort S., Ballmick A., Tan Y., Betancourt M., Viteri E., Ramkhellawan G.C., Ewald E. (2013). Infiltration of proinflammatory m1 macrophages into the outer retina precedes damage in a mouse model of age-related macular degeneration. Int. J. Inflam..

[5] Huang Z., Zhou T., Sun X., Zheng Y., Cheng B., Li M., Liu X., He C. (2018). Necroptosis in microglia contributes to neuroinflammation and retinal degeneration through TLR4 activation. Cell Death Differ..

[6] Dunaief J.L., Dentchev T., Ying G.-S., Milam A.H. (2002). The role of apoptosis in age-related macular degeneration. Arch. Ophthalmol..

[7] Sanges D., Comitato A., Tammaro R., Marigo V. (2006). Apoptosis in retinal degeneration involves cross-talk between apoptosis-inducing factor (AIF) and caspase-12 and is blocked by calpain inhibitors. Proc. Natl. Acad. Sci. USA.

[8] Viringipurampeer I.A., Shan X., Gregory-Evans K., Zhang J.P., Mohammadi Z., Gregory-Evans C.Y. (2014). Rip3 knockdown rescues photoreceptor cell death in blind pde6c zebrafish. Cell Death Differ..

[9] Brent G.A. (2012). Mechanisms of thyroid hormone action. J. Clin. Investig..

[10] Cheng S.Y., Leonard J.L., Davis P.J. (2010). Molecular aspects of thyroid hormone actions. Endocr. Rev..

[11] Forrest D., Reh T.A., Rusch A. (2002). Neurodevelopmental control by thyroid hormone receptors. Curr. Opin. Neurobiol..

[12] Yang F., Ma H., Boye S.L., Hauswirth W.W., Ding X.-Q. (2018). Overexpression of Type 3 Iodothyronine Deiodinase Reduces Cone Death in the Leber Congenital Amaurosis Model Mice. Adv. Exp. Med. Biol..

[13] Yang F., Ma H., Butler M.R., Ding X.-Q. (2018). Deficiency of type 2 iodothyronine deiodinase reduces necroptosis activity and oxidative stress responses in retinas of Leber congenital amaurosis model mice. FASEB J..

[14] Ma H., Thapa A., Morris L., Redmond T.M., Baehr W., Ding X.-Q. (2014). Suppressing thyroid hormone signaling preserves cone photoreceptors in mouse models of retinal degeneration. Proc. Natl. Acad. Sci. USA.

[15] Ma H.W., Yang F., Butler M.R., Belcher J., Redmond T.M., Placzek A.T., Scanlan T.S., Ding X. (2017). Inhibition of thyroid hormone receptor locally in the retina is a therapeutic strategy for retinal degeneration. FASEB J..

[16] Yang F., Ma H., Belcher J., Butler M.R., Redmond T.M., Boye S.L., Hauswirth W.W., Ding X. (2016). Targeting iodothyronine deiodinases locally in the retina is a therapeutic strategy for retinal degeneration. FASEB J..

[17] Ma H., Yang F., Ding X.Q. (2020). Inhibition of thyroid hormone signaling protects retinal pigment epithelium and photoreceptors from cell death in a mouse model of age-related macular degeneration. Cell Death Dis..

[18] Ma H., Yang F., Ding X.Q. (2022). Deficiency of thyroid hormone receptor protects retinal pigment epithelium and photoreceptors from cell death in a mouse model of age-related macular degeneration. Cell Death Dis..

[19] Ma H., Yang F., York L.R., Li S., Ding X.-Q. (2023). Excessive Thyroid Hormone Signaling Induces Photoreceptor Degeneration in Mice. eNeuro.

[20] Ng L., Lyubarsky A., Nikonov S.S., Ma M., Srinivas M., Kefas B., Germain D.L.S., Hernandez A., Pugh E.N., Forrest D. (2010). Type 3 deiodinase, a thyroid-hormone-inactivating enzyme, controls survival and maturation of cone photoreceptors. J. Neurosci..

[21] Li S., Ma H., Ding X.Q. (2025). Resveratrol Protects Photoreceptors in Mouse Models of Retinal Degeneration. Antioxidants.

[22] Chaker L., Buitendijk G.H., Dehghan A., Medici M., Hofman A., Vingerling J.R., Franco O.H., Klaver C.C., Peeters R.P. (2015). Thyroid function and age-related macular degeneration: A prospective population-based cohort study—The Rotterdam Study. BMC Med..

[23] Gopinath B., Liew G., Kifley A., Mitchell P. (2016). Thyroid Dysfunction and Ten-Year Incidence of Age-Related Macular Degeneration. Investig. Ophthalmol. Vis. Sci..

[24] Age-Related Eye Disease Study Research Group (2000). Risk factors associated with age-related macular degeneration. A case-control study in the age-related eye disease study: Age-Related Eye Disease Study Report Number 3. Ophthalmology.

[25] Lin S.Y., Hsu W.-H., Lin C.-L., Lin C.-C., Lin J.-M., Chang Y.-L., Hsu C.-Y., Kao C.-H. (2018). Evidence for an Association between Macular Degeneration and Thyroid Cancer in the Aged Population. Int. J. Environ. Res. Public Health.

[26] Chatziralli I., MMitropoulos P.G., Niakas D., Labiris G. (2017). Thyroidopathy and Age-Related Macular Degeneration: Is There Any Correlation. Biomed. Hub.

[27] Hung S.H., Xirasagar S., Kuang T.-M.T., Chang W.-W., Cheng Y.-F., Kuo N.-W., Lin H.-C. (2022). Association of Age-Related Macular Degeneration with Prior Hyperthyroidism and Hypothyroidism: A Case-Control Study. J. Pers. Med..

[28] Xu Z., Zhang M., Zhang Q., Xu T., Tao L. (2021). Thyroid Disease Is Associated with Higher Age-Related Macular Degeneration Risk: Results from a Meta-Analysis of Epidemiologic Studies. Ophthalmic Res..

[29] Farvardin M., Mousavi S.E., Zare K., Bazdar S., Farvardin Z., Johari M. (2021). Thyroid Dysfunction as a Modifiable Risk Factor for Wet Type Age-Related Macular Degeneration: A Case-Control Study. J. Curr. Ophthalmol..

[30] Li X., Cheng J., Wang M., Zhong Y., Shi G., Yu A.-Y. (2022). Causal Associations of Thyroid Function and Age-Related Macular Degeneration: A Two-Sample Mendelian Randomization Study. Am. J. Ophthalmol..

[31] Abdelkader M., Abass N. (2019). The Relation Between Age Related Macular Degeneration and Thyroid Disorders. Int. J. Ophthalmol. Vis. Sci..

[32] Blum Meirovitch S., Leibovitch I., Kesler A., Varssano D., Rosenblatt A., Neudorfer M. (2017). Retina and Nerve Fiber Layer Thickness in Eyes with Thyroid-Associated Ophthalmopathy. Isr. Med. Assoc. J..

[33] Sayin O., Yeter V., Ariturk N. (2016). Optic Disc, Macula, and Retinal Nerve Fiber Layer Measurements Obtained by OCT in Thyroid-Associated Ophthalmopathy. J. Ophthalmol..

[34] Ceresini G., Lauretani F., Maggio M., Ceda G.P., Morganti S., Usberti E., Chezzi C., Valcavi R., Bandinelli S., Guralnik J.M. (2009). Thyroid function abnormalities and cognitive impairment in elderly people: Results of the Invecchiare in Chianti study. J. Am. Geriatr. Soc..

[35] Kalmijn S., Mehta K.M., Pols H.A.P., Hofman A., Drexhage H.A., Breteler M.M.B. (2000). Subclinical hyperthyroidism and the risk of dementia. The Rotterdam study. Clin. Endocrinol..

[36] Cho Y.Y., Kim B., Shin D.W., Youn J., Mok J.O., Kim C.-H., Kim S.W., Chung J.H., Han K., Kim T.H. (2022). Graves’ disease and the risk of Parkinson’s disease: A Korean population-based study. Brain Commun..

[37] Vandenabeele P., Galluzzi L., Vanden Berghe T.V., Kroemer G. (2010). Molecular mechanisms of necroptosis: An ordered cellular explosion. Nat. Rev. Mol. Cell Biol..

[38] Newton K. (2015). RIPK1 and RIPK3: Critical regulators of inflammation and cell death. Trends Cell Biol..

[39] Paudel S., Jeyaseelan S. (2021). Kill Two Birds with One Stone: Role of the RIPK-3 in Necroptosis and Inflammasome Activation. Am. J. Respir. Cell Mol. Biol..

[40] Moriwaki K., Balaji S., McQuade T., Malhotra N., Kang J., Chan F.K.-M. (2014). The necroptosis adaptor RIPK3 promotes injury-induced cytokine expression and tissue repair. Immunity.

[41] Afonso M.B., Islam T., Magusto J., Amorim R., Lenoir V., Simões R.F., Teixeira J., Silva L.C., Wendum D., Jéru I. (2023). RIPK3 dampens mitochondrial bioenergetics and lipid droplet dynamics in metabolic liver disease. Hepatology.

[42] Mandal P., Berger S.B., Pillay S., Moriwaki K., Huang C., Guo H., Lich J.D., Finger J., Kasparcova V., Votta B. (2014). RIP3 induces apoptosis independent of pronecrotic kinase activity. Mol. Cell.

[43] York L., Ma H., Yang F., Primeaux C., Griffin C., Ding X.Q. The Role of RIPK3 Signaling in Thyroid Hormone-Induced Photoreceptor Degeneration. Proceedings of the 2023 ARVO Annual Meeting.

[44] Wang H., Sun L., Su L., Rizo J., Liu L., Wang L.-F., Wang F.-S., Wang X. (2014). Mixed lineage kinase domain-like protein MLKL causes necrotic membrane disruption upon phosphorylation by RIP3. Mol. Cell.

[45] Liu S., Liu H., Johnston A., Hanna-Addams S., Reynoso E., Xiang Y., Wang Z. (2017). MLKL forms disulfide bond-dependent amyloid-like polymers to induce necroptosis. Proc. Natl. Acad. Sci. USA.

[46] Cai Z., Jitkaew S., Zhao J., Chiang H.-C., Choksi S., Liu J., Ward Y., Wu L.-G., Liu Z.-G. (2014). Plasma membrane translocation of trimerized MLKL protein is required for TNF-induced necroptosis. Nat. Cell Biol..

[47] Wu J., Huang Z., Ren J., Zhang Z., He P., Li Y., Ma J., Chen W., Zhang Y., Zhou X. (2013). Mlkl knockout mice demonstrate the indispensable role of Mlkl in necroptosis. Cell Res..

[48] Sun L., Wang H., Wang Z., He S., Chen S., Liao D., Wang L., Yan J., Liu W., Lei X. (2012). Mixed lineage kinase domain-like protein mediates necrosis signaling downstream of RIP3 kinase. Cell.

[49] Murakami Y., Matsumoto H., Roh M., Suzuki J., Hisatomi T., Ikeda Y., Miller J.W., Vavvas D.G. (2012). Receptor interacting protein kinase mediates necrotic cone but not rod cell death in a mouse model of inherited degeneration. Proc. Natl. Acad. Sci. USA.

[50] Trichonas G., Murakami Y., Thanos A., Morizane Y., Kayama M., Debouck C.M., Hisatomi T., Miller J.W., Vavvas D.G. (2010). Receptor interacting protein kinases mediate retinal detachment-induced photoreceptor necrosis and compensate for inhibition of apoptosis. Proc. Natl. Acad. Sci. USA.

[51] Moriwaki K., Chan F.K. (2014). Necrosis-dependent and independent signaling of the RIP kinases in inflammation. Cytokine Growth Factor Rev..

[52] Li D., Chen J., Guo J., Li L., Cai G., Chen S., Huang J., Yang H., Zhuang Y., Wang F. (2021). A phosphorylation of RIPK3 kinase initiates an intracellular apoptotic pathway that promotes prostaglandin(2alpha)-induced corpus luteum regression. Elife.

[53] Newton K., Zhang J., Wang Z., Li T., Liu C., Kang X., Cui X., Yang J., Qu H., Duanmu J. (2014). Activity of protein kinase RIPK3 determines whether cells die by necroptosis or apoptosis. Science.

[54] Zhang W., Zhang J., Wang Z., Li T., Liu C., Kang X., Cui X., Yang J., Qu H., Duanmu J. (2024). Extracellular RIPK3 Acts as a Damage-Associated Molecular Pattern to Exaggerate Cardiac Ischemia/Reperfusion Injury. Circulation.

[55] Zhao X., Quan J., Tan Y., Liu Y., Liao C., Li Z., Liao W., Liu J., Cao Y., Luo X. (2021). RIP3 mediates TCN-induced necroptosis through activating mitochondrial metabolism and ROS production in chemotherapy-resistant cancers. Am. J. Cancer Res..

[56] Sureshbabu A., Patino E., Ma K.C., Laursen K., Finkelsztein E.J., Akchurin O., Muthukumar T., Ryter S.W., Gudas L., Choi A.M.K. (2018). RIPK3 promotes sepsis-induced acute kidney injury via mitochondrial dysfunction. JCI Insight.

[57] Ma H., Stanford D., Freeman W.M., Ding X.-Q. (2024). Transcriptomic Analysis Reveals That Excessive Thyroid Hormone Signaling Impairs Phototransduction and Mitochondrial Bioenergetics and Induces Cellular Stress in Mouse Cone Photoreceptors. Int. J. Mol. Sci..

[58] Moriwaki K., Bertin J., Gough P.J., Chan F.K.-M. (2015). A RIPK3-caspase 8 complex mediates atypical pro-IL-1beta processing. J. Immunol..

[59] Orozco S.L., Daniels B.P., Yatim N., Messmer M.N., Quarato G., Chen-Harris H., Cullen S.P., Snyder A.G., Ralli-Jain P., Frase S. (2019). RIPK3 Activation Leads to Cytokine Synthesis that Continues after Loss of Cell Membrane Integrity. Cell Rep..

[60] Sharma D., Malik A., Balakrishnan A., Malireddi R.K.S., Kanneganti T.-D. (2020). RIPK3 Promotes Mefv Expression and Pyrin Inflammasome Activation via Modulation of mTOR Signaling. J. Immunol..

[61] Olivares-Gonzalez L., Velasco S., Campillo I., Rodrigo R. (2021). Retinal Inflammation, Cell Death and Inherited Retinal Dystrophies. Int. J. Mol. Sci..

[62] Lai D., Wu Y., Shao C., Qiu Q. (2023). The Role of Muller Cells in Diabetic Macular Edema. Investig. Ophthalmol. Vis. Sci..

[63] Jahnke L., Zandi S., Elhelbawi A., Conedera F.M., Enzmann V. (2023). Characterization of Macroglia Response during Tissue Repair in a Laser-Induced Model of Retinal Degeneration. Int. J. Mol. Sci..

[64] Ng L., Liu H., Liu Y., Forrest D. (2023). Biphasic expression of thyroid hormone receptor TRbeta1 in mammalian retina and anterior ocular tissues. Front. Endocrinol..

[65] Liu Y., Ng L., Liu H., Heuer H., Forrest D. (2024). Cone photoreceptor differentiation regulated by thyroid hormone transporter MCT8 in the retinal pigment epithelium. Proc. Natl. Acad. Sci. USA.

[66] Ding X.-Q., Yang F., Butler M., Malek G., Ma H. (2019). Thyroid Hormone Regulation of Retinal Pigment Epithelium Morphology and Survival. Investig. Ophthalmol. Vis. Sci..

[67] Murphy J.M., Czabotar P.E., Hildebrand J.M., Lucet I.S., Zhang J.-G., Alvarez-Diaz S., Lewis R., Lalaoui N., Metcalf D., Webb A.I. (2013). The pseudokinase MLKL mediates necroptosis via a molecular switch mechanism. Immunity.

[68] Colijn S., Gao S., Ingram K.G., Menendez M., Muthukumar V., Silasi-Mansat R., Chmielewska J.J., Hinsdale M., Lupu F., Griffin C.T. (2020). The NuRD chromatin-remodeling complex enzyme CHD4 prevents hypoxia-induced endothelial Ripk3 transcription and murine embryonic vascular rupture. Cell Death Differ..

[69] Le Y.Z., Zheng L., Zheng W., Ash J.D., Agbaga M.-P., Zhu M., E Anderson R. (2006). Mouse opsin promoter-directed Cre recombinase expression in transgenic mice. Mol. Vis..

[70] Le Y.Z., Ash J.D., Al-Ubaidi M.R., Chen Y., Ma J.-X., Anderson R.E. (2004). Targeted expression of Cre recombinase to cone photoreceptors in transgenic mice. Mol. Vis..

[71] Hamidi S., Aliesky H., Chen C.-R., Rapoport B., McLachlan S.M. (2010). Variable suppression of serum thyroxine in female mice of different inbred strains by triiodothyronine administered in drinking water. Thyroid.

