# Supplementary material for: RIPK3 Contributes to Thyroid Hormone-Induced Photoreceptor Degeneration
Source: Int J Mol Sci. 2025 Aug 22;26(17):8154. doi: 10.3390/ijms26178154 (PMC12428710; doi:10.3390/ijms26178154)
Supplement: Supplementary file 1 [file ijms-26-08154-s001.zip › ijms-3765527-supplementary.pdf]

For consideration of publication in International Journal of Molecular Sciences

RIPK3 Contributes to Thyroid Hormone-Induced Photoreceptor Degeneration

Lilliana R. York [1], Hongwei Ma [1], Yun Le [2], Courtney T. Griffin [1,3] and Xi-Qin Ding [1]*

[1] Department of Cell Biology, University of Oklahoma Health Sciences, Oklahoma City, Oklahoma, 73104, USA
[2] Departments of Medicine Endocrinology, Cell Biology, and Ophthalmology, and Harold Hamm Diabetes Center, University of Oklahoma Health Sciences, Oklahoma City, Oklahoma, 73104, USA
[3] Cardiovascular Biology Research Program, Oklahoma Medical Research Foundation, Oklahoma City, Oklahoma, 73104, USA

* Correspondence: xi-qin-ding@ou.edu

Supplementary Figures

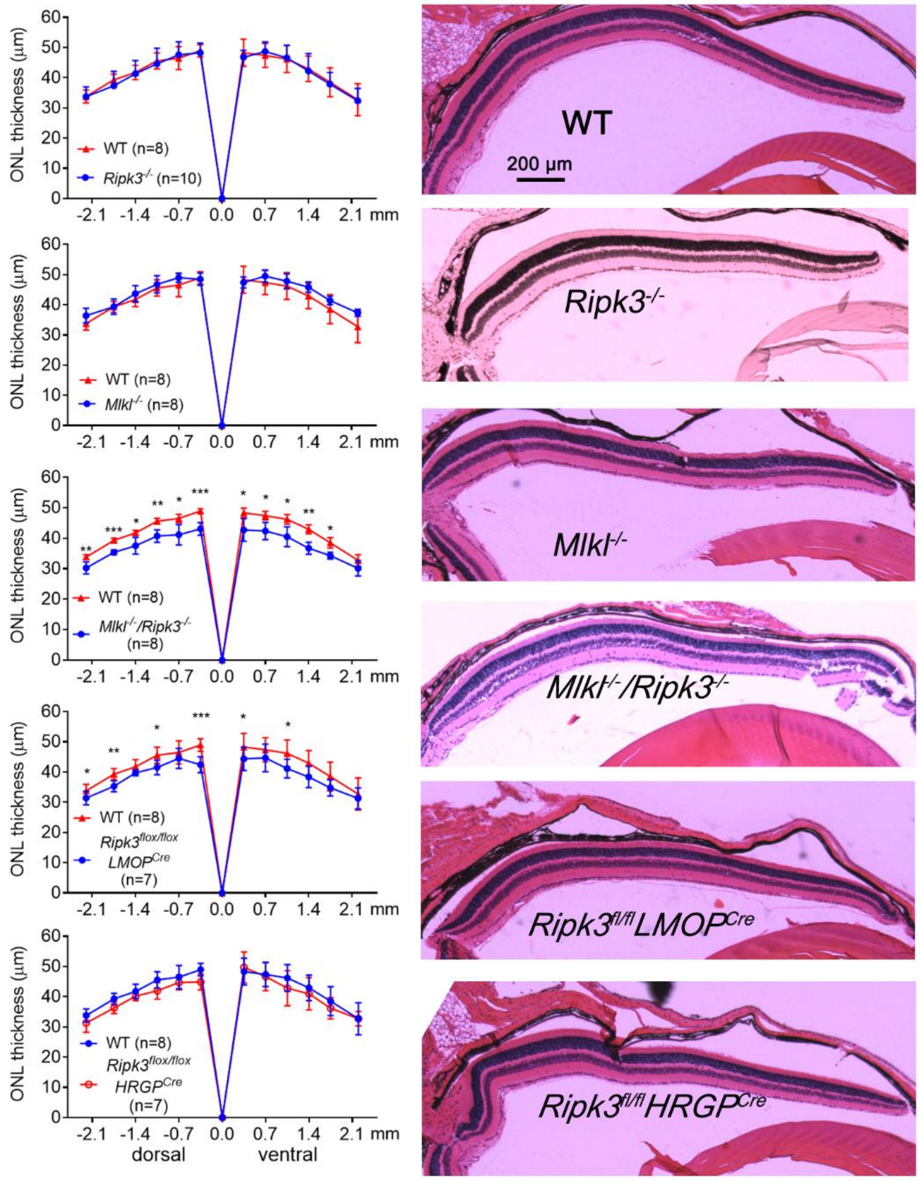

Supplementary Figure S1. ONL thickness and retinal morphology in wild-type and mutant mice under untreated condition. **A.** Shown are quantitative analysis of ONL thickness in C57BL/6, *Ripk3*$^{-/-}$, *Mlkl*$^{-/-}$, *Mlkl*$^{-/-}$/*Ripk3*$^{-/-}$, *Ripk3*$^{flox/flox}$/*LMOP*^{Cre}, and *Ripk3*$^{flox/flox}$/*HRGP*^{Cre} mice at one month of age. Data are presented as means ± SD for 7-10 mice per group. Unpaired Student's *t*-test/Mann-Whitney test was used to test for differences between two groups of data (**p* < 0.05, ***p* < 0.01, ****p* <0.001, compared with wild-type mice). **B.** Shown are the whole dorsal areas of retinal images of H&E-staining in the wild-type and mutant mice.

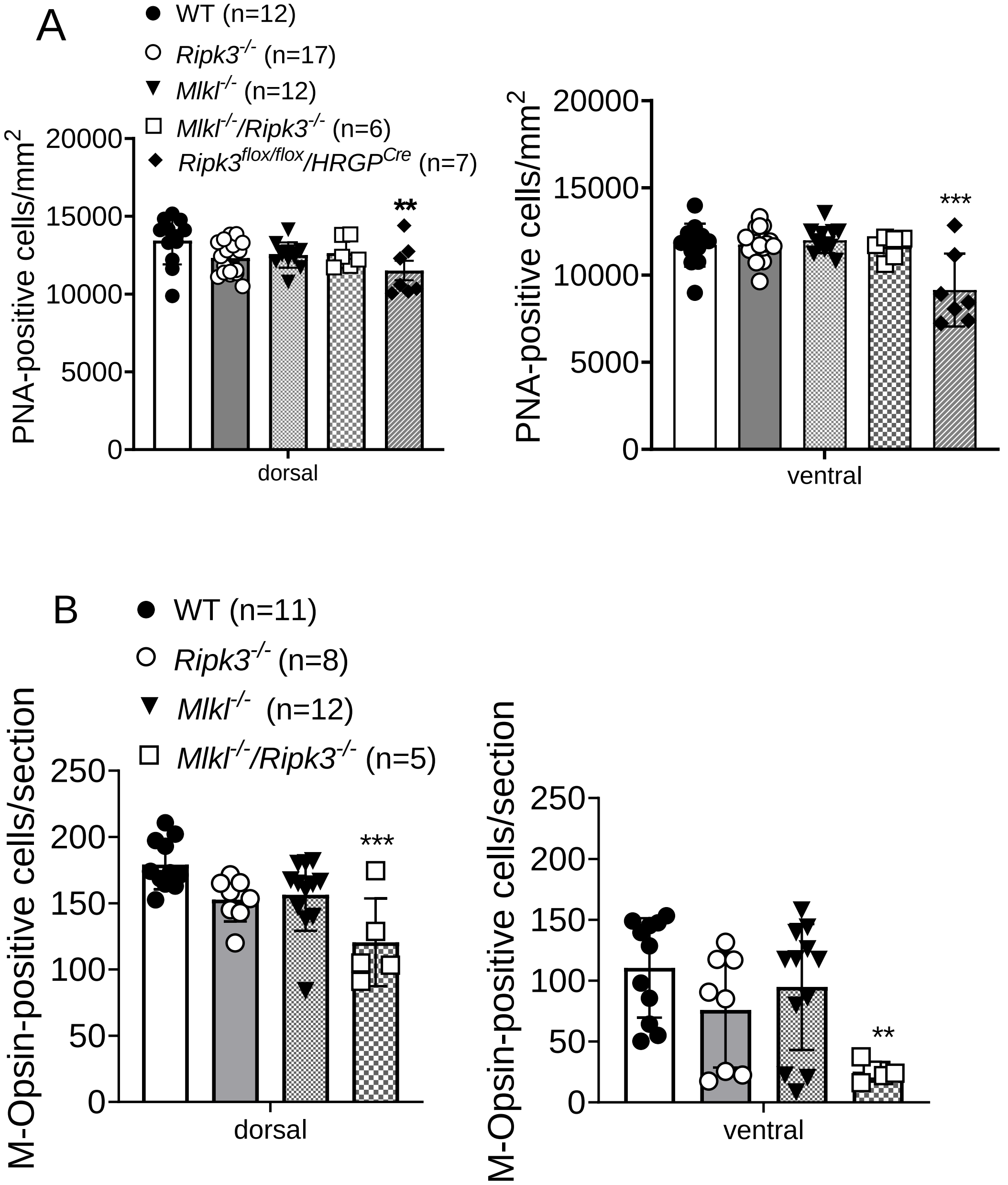

Supplementary Figure S2. Cone density evaluation in wild-type and mutant mice under untreated condition. **A.** Shown are quantitative analysis of cone density evaluated by PNA labeling of retinal whole-mounts in C57BL/6, *Ripk3*$^{-/-}$, *Mlkl*$^{-/-}$, *Mlkl*$^{-/-}$/*Ripk3*$^{-/-}$, and *Ripk3*$^{flox/flox}$/*HRGP*^{Cre} mice at one month of age. **A.** Shown are quantitative analysis of cone density evaluated by M-opsin labeling of retinal cross-sections in C57BL/6, *Ripk3*$^{-/-}$, *Mlkl*$^{-/-}$, and *Mlkl*$^{-/-}$/*Ripk3*$^{-/-}$ mice at one month of age. Data are presented as means ± SD for 5-17 mice per group. One-way ANOVA was used for significance within sets of data, followed by Dunnett's multiple comparisons test (*p < 0.05, **p < 0.01, ***p <0.001, compared with wild-type mice).

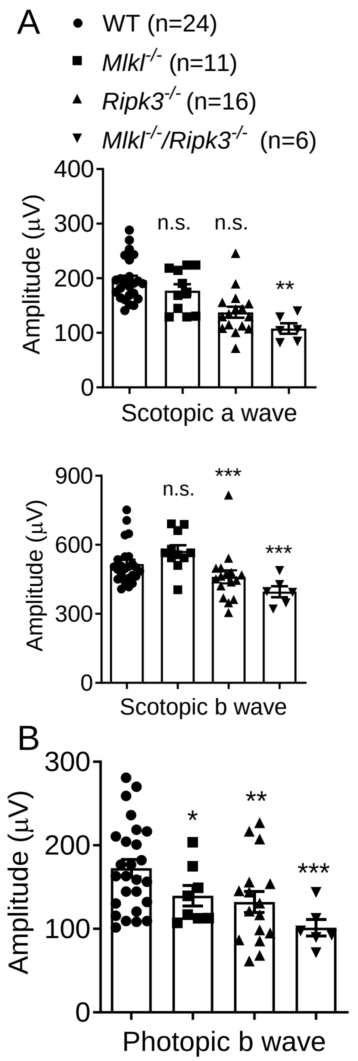

Supplementary Figure S3. ERG responses in wild-type and mutant mice under untreated condition. Shown are scotopic (**A**) and photopic (**B**) ERG responses in C57BL/6, *Ripk3*$^{-/-}$, *Mlkl*$^{-/-}$, and *Mlkl*$^{-/-}$/*Ripk3*$^{-/-}$ mice at one month of age. Data are represented as means ± SD for 6-24 mice per group. One-way ANOVA was used for significance within sets of data, followed by Dunnett's multiple comparisons test (*$p < 0.05$, **$p < 0.01$, ***p <0.001, compared with wild- type mice).
